# Insulin pump settings and glucose patterns during a 1008-km non-stop bicycle race in a patient with type 1 diabetes mellitus

**DOI:** 10.1007/s00592-018-1254-4

**Published:** 2018-11-15

**Authors:** Tomasz Klupa, Jerzy Hohendorff, Teresa Benbenek-Klupa, Bartłomiej Matejko, Maciej T. Malecki

**Affiliations:** 1 Department of Metabolic Diseases, Jagiellonian University Medical College, 15 Kopernika Street, 31-501 Kraków, Poland; 2 University Hospital, Kraków, Poland; 3 DiabWay Educational Enterprise, Kraków, Poland

There is a general consensus that physical activity should be recommended for individuals with type 1 diabetes mellitus (T1DM) [1]. While moderate exercise can be relatively easily managed with respect to diabetes, extreme or prolonged sport activity still remains a challenge [1]. Extreme sports are in general not recommended for individuals with T1DM, but more and more patients choose them. In such cases, diabetes management should be highly individualized, and advanced technologies often can be useful [1].

Here we describe the 1008-km non-stop bicycle race successfully completed by a 44-year-old individual with 22-year duration of T1DM. The patient is physically very active and enjoys extreme challenges. His clinical characteristics have been presented before [2]. He previously captured Aconcagua mountain [2], participated in Damavand expedition [3], and completed a trail running ultramarathon, to list only few of his achievements. His last available HbA1c determined 2 months before competition was 6.8% (50.8 mmol/mol), current weight 78 kg, and BMI 23.8 kg/m^2^. Informed consent about using his personal and medical data was obtained before the race.

The patient uses The MiniMed 640G system (Medtronic, Inc., Northridge, CA, USA) which includes a feature called SmartGuard™ that suspends insulin delivery when the glucose value is predicted to reach or fall below a preset low glucose limit within 30 min and automatically restarts basal insulin on recovery from hypoglycemia [4]. Settings for suspend before low are customizable. Since the patient was switched to a 640G personal insulin pump, his Smart Guard™ feature was set to 3.3–3.9 mmol/L (60–70 mg/dL). This target was successfully tested in clinical trials and is supposed to be effective for glucose management for majority of individuals with T1DM [5]. However, to the author’s knowledge it has been never checked under conditions of prolonged and extreme exercise.

The Baltic Sea–Bieszczady Mountains bicycle race took part during August 24th–27th, the time limit to complete the 1008-km race was 70 h. The most important challenge with respect to glucose management was choosing the proper pump settings to successfully prevent hypoglycemia, keep blood glucose concentration in a range optimal for prolonged aerobic exercise and allow only minimal intervention from patient side.

The first question to be addressed before setting the SmartGuard™ feature was what should be the target glucose levels for heavy, aerobic, 70-h continuous exercise. Based on consensus published recently, a reasonable starting range for most patients doing aerobic exercise is 7–10 mmol/L (126–180 mg/dL), higher concentrations might be advised in some situations where added protection against hypoglycaemia is needed [1]. The maintenance of a concentration of about 6–8 mmol/L (108–144 mg/dL) may be ideal [1]. Our previous experience from trail ultramarathon pointed, however, that SmartGuard™ feature setting at 6.9 mmol/L (70 mg/dL) is too low to keep the glucose level during prolonged exercise in target. Exercise induces insulin-independent glucose uptake into muscle [1]. An extended duration of exercise leads to decreased reliance on muscle glycogen as fuel and increased reliance on lipid oxidation and glucose derived from plasma [1]. Plasma insulin concentrations must be kept low, otherwise the rise in counter-regulatory hormones will not be effective enough in the promotion of hepatic glucose production [1]. One of the priorities during prolonged exercise is avoidance of hypoglycemia since each hypoglycemic episode decreases patients’ performance significantly and in our case of extremely long exercise could result in race discontinuation [1]. This is why we decided to set SmartGuard™ option at 90 mg/dL for the whole race. Patient successfully completed the race within 68 h and 4 min in good shape.

During 3-day race, patient’s mean glucose patterns were as follows:

- Day 1: mean: 10 mmol/L (180 mg/dL), SD: 3.9 mmol/L (70 mg/dL), time in target 45%, time > target 33%, time < target 23% (target 3.9–10.0 mmol/mol (70–180 mg/dL)), total daily insulin dose: 20, 83 IU (72% delivered as basal), total time of basal insulin delivery suspension: 6 h 41 min.
- Day 2: mean: 11.3 mmol/L (204 mg/dL), SD: 4.4 mmol/L (80 mg/dL), time in target 62%, time > target 38%, time < target 0%, total daily insulin dose: 26, 4 IU (65% delivered as basal), total basal insulin delivery suspension: 4 h 20 min.
- Day 3: mean: 11 mmol/L (198 mg/dL), SD: 4.6 mmol/L (83 mg/dL), time in target 60%, time > target 40%, time < target 0%, total daily insulin dose: 30,68 IU (59% delivered as basal), total basal insulin delivery suspension: 4 h 21 min.

**Fig. 1:**
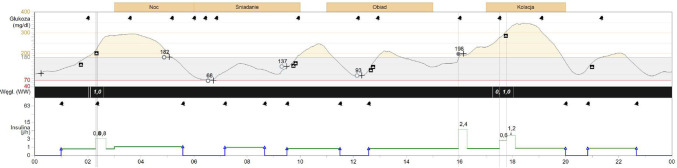
The download of the patients’ 640G insulin pump (hours 4–28)

Figure 1 shows the insulin pump download from one of the race days (hours 4–28).

Insulin pump settings and patient’s glucose management resulted in very effective hypoglycemia prevention. The individual did not have to manually stop or reduce basal insulin. The mean glucose concentration as the number of hyperglycemic excursions should be considered to high. This is probably the result of underbolusing—the total dose of insulin delivered as boluses was only 28.2 IU over the 3-day race. In addition, hyperglycemic excursions could have also resulted from having few large meals not effectively covered with insulin boluses instead of multiple meals and snacks. This was the patient’s choice with the goal to save time during competition. Although there were many hours of suspension during the race, we think threshold for suspension was set correctly to successfully prevent hypoglycemia which was the main target.

To conclude, completing the 1008-km non-stop bike race was a phenomenal achievement for a patient with T1DM. Setting “suspend before low” target at 5 mmol/L (90 mg/dL) allowed the patient to avoid hypoglycemia very effectively. However, the performance could be probably even better with a lower mean glucose level, which could have been achieved with higher meal boluses and/or multiple smaller meals/snack rather than few large meals.
